# Arabidopsis molybdenum cofactor sulfurase ABA3 contributes to anthocyanin accumulation and oxidative stress tolerance in ABA-dependent and independent ways

**DOI:** 10.1038/s41598-018-34862-1

**Published:** 2018-11-09

**Authors:** Shunsuke Watanabe, Muneo Sato, Yuji Sawada, Maho Tanaka, Akihiro Matsui, Yuri Kanno, Masami Yokota Hirai, Motoaki Seki, Atsushi Sakamoto, Mitsunori Seo

**Affiliations:** 1 RIKEN Center for Sustainable Resource Science, 1-7-22 Suehiro-cho, Tsurumi-ku, Yokohama, Kanagawa 230-0045 Japan; 2 RIKEN Cluster for Pioneering Research, 2-1 Hirosawa, Wako, Saitama 351-0198 Japan; 3 Department of Mathematics and Life Sciences, Graduate School of Science, Hiroshima University, 1-3-1 Kagamiyama, Higashi-Hiroshima, 739-8526 Japan

## Abstract

Arabidopsis ABA3 is an enzyme involved in the synthesis of the sulfurated form of the molybdenum (Mo) cofactor (MoCo), which is required for the enzymatic activity of so-called Mo enzymes such as aldehyde oxidase (AO) and xanthine dehydrogenase (XDH). It has been reported that AO and XDH are essential for the biosynthesis of the bioactive compounds, ABA and allantoin, respectively. However, *aba3* mutants often exhibit pleiotropic phenotypes that are not explained by defects in ABA and/or allantoin biosynthesis, leading us to hypothesize that ABA3 regulates additional metabolic pathways. To reveal the currently unidentified functions of ABA3 we compared transcriptome and metabolome of the Arabidopsis *aba3* mutant with those of wild type and a typical ABA-deficient mutant *aba2*. We found that endogenous levels of anthocyanins, members of the flavonoid group, were significantly lower in the *aba3* mutant than in the wild type or the *aba2* mutant under oxidative stress. In contrast, mutants defective in the AO and XDH holoenzymes accumulated significantly higher levels of anthocyanins when compared with *aba3* mutant under the same conditions. Our findings shed light on a key role of ABA3 in the ABA- and allantoin-independent accumulation of anthocyanins during stress responses.

## Introduction

Molybdenum (Mo) is an essential metal for nearly all organisms because it is incorporated into pterin-based cofactors (Mo cofactor; MoCo) and functions as the catalytic center of enzymes involved in fundamental metabolic processes^1^. Deficiencies in MoCo result in severe pleiotropic phenotypes in both plants and animals^2–6^, indicating that MoCo, and hence Mo enzymes, play important roles in a wide range of biochemical and physiological processes.

**Figure 1:**
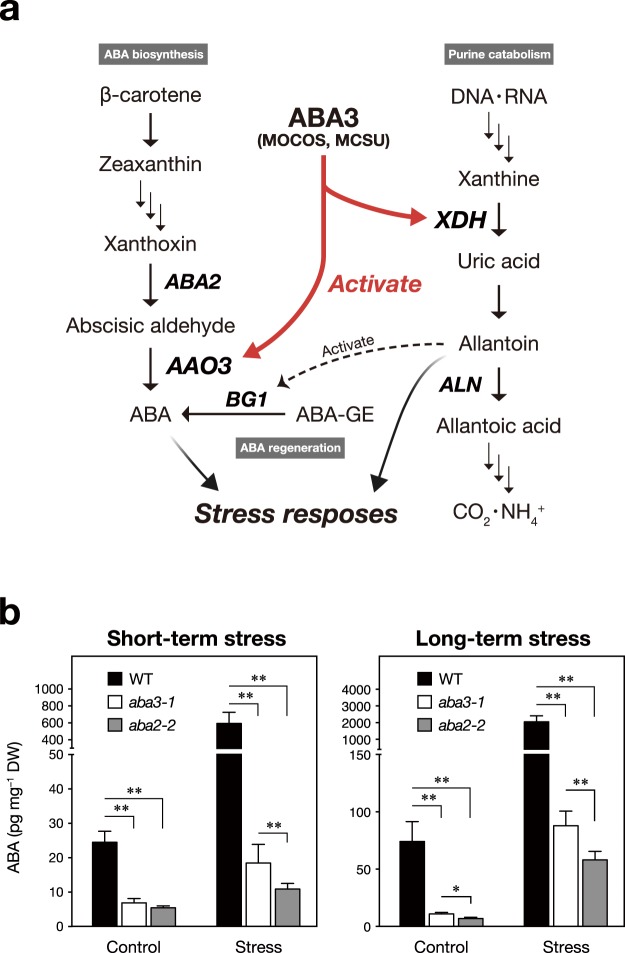
Physiological functions of the Arabidopsis ABA3 enzyme in stress responses. (**a**) A schematic illustration of the ABA3-regulated metabolic pathways. (**b**) Endogenous ABA levels in wild-type (WT), *aba3-1* and *aba2-2* plants under short-term and long-term stress conditions. Values are means (±SD) of five biological replicates (^*^P < 0.01, ^**^P < 0.001 by Tukey’s multiple comparison test).

The main pathway of MoCo biosynthesis is conserved across kingdoms^1^. It starts with the production of metal-binding pterin (MPT) from GTP and then Mo is inserted into MPT to form MoCo^7,8^. Among more than 40 Mo enzymes identified to date, four types of enzymes, nitrate reductase (NR)^9^, sulfite oxidase (SO)^10^, xanthine oxidase/dehydrogenase (XO/XDH)^11^, and aldehyde oxidase (AO)^12^, have been well characterized in plants. More recently, orthologous genes for mitochondrial amidoxime reducing component (mARC), which was identified as a Mo-containing enzyme in pig liver, were found in the Arabidopsis genome^13,14^. Based on the chemical structures of MoCos, these Mo enzymes are classified into two groups: the NR/SO/mARC family, whose Mo center forms a covalent bond with a strictly conserved cysteine residue, and the XDH/AO family, whose Mo forms a double bond with a terminal sulfur using a free cysteine as the sulfur donor. The modification reaction allowing MoCo to be utilized for AO and XDH is catalyzed by MoCo sulfurase (MOCOS or MCSU) (Fig. 1a).

It has been suggested that AO plays important roles in the detoxification of xenobiotics, such as drugs and pollutants, in animals^15–17^. In contrast, the involvement of plant AOs in response to environmental changes, including various stresses, has been well documented. For example, it was predicted that the *de novo* biosynthesis of the phytohormone ABA requires AOs from observations that mutants lacking AO activities, due to MoCo deficiencies, contained reduced endogenous ABA levels^18,19^. In Arabidopsis, the ABA-deficient *aba3* mutant was originally isolated based on its ability to germinate in the presence of the gibberellin (GA) biosynthesis inhibitor paclobutrazol^20^, and subsequent studies showed that the causal gene (*ABA3*) encodes MOCOS^21,22^. There are four genes encoding AOs in Arabidopsis (*AAO1–4*)^23^. It has been demonstrated that the AAO3 enzyme catalyzes the oxidation of abscisic aldehyde to produce ABA^24,25^, and a mutation in the *AAO3* gene resulted in reduction of endogenous ABA levels with increased transpiration rates and lower resistance to water shortages^24,25^. In contrast, the AAO1 enzyme was reported to be involved in the metabolism of an indolic compound, camalexin, which is thought to contribute to defense responses against pathogen infection^26^. It has also been reported recently that the AAO4 enzyme plays an important role in silique senescence, although the substrate(s) of the enzyme is unknown^27^.

Studies on leguminous plants such as soybean revealed that XDH is an essential enzyme in the catabolism of purine compounds synthesized from ammonium produced by root nodule bacteria to form nitrogen-rich metabolites, such as allantoin and allantoic acid, that can be translocated to sink tissues and utilized as major nitrogen sources^28^. RNA interference targeting of two Arabidopsis *XDH* genes (*AtXDH1* and *AtXDH2*) caused typical symptoms of nitrogen deficiency, including growth retardation, early senescence, and reduced fertility, indicating that purine catabolism mediated by XDH also plays important roles in nitrogen recycling in non-leguminous plants^29^. It has also been reported that XDH plays important roles in response to prolonged dark periods^30^, water deficit^31^ and pathogen attack^32^. These functions of XDH can be explained partly by the activities of allantoin to scavenge reactive oxygen species (ROS)^30,33^ and/or to activate the β-glucosidase BGLU18 enzyme that catalyzes the hydrolysis of inactive ABA conjugates (ABA-glucose ester) to produce free bioactive ABA^34,35^.

Consistent with the roles of Mo enzymes in multiple aspects of plant life, mutants defective in MOCOS, such as Arabidopsis *aba3*, show pleiotropic phenotypes^36^. Interestingly, it is also true, however, that the phenotypes observed in the mutants are not always explained by the loss of AO and/or XDH functions. For example, the expression of several stress responsive genes was differentially regulated between *aba3* and another ABA-deficient mutant, *aba1*^21^. It has also been reported that the *aba3* mutant is impaired in chloroplast protein import^37^. These observations suggest that the MOCOS enzyme has uncharacterized functions. Since MOCOS is essential for activating several Mo enzymes, we hypothesized that it might be involved in the metabolism of currently unidentified bioactive compounds.

In this study, we conducted comparative analyses of the transcriptome and metabolome using the Arabidopsis *aba3* mutant and a typical ABA-deficient mutant *aba2*. *ABA2* encodes a short-chain dehydrogenase/reductase (SDR) which catalysis the conversion of xanthoxin to abscisic aldehyde. Like ABA3, ABA2 is encoded by singe gene in Arabidopsis. However, the functions of ABA2 other than ABA biosynthesis have not been proposed. We revealed that anthocyanin biosynthesis was differentially regulated between the two mutants. Possible roles of MOCOS in ABA- and allantoin-independent stress responses are discussed.

## Results

### ABA-dependent and -independent transcriptional responses to osmotic stress mediated by Arabidopsis MOCOS ABA3

It has been reported that the Arabidopsis MOCOS (ABA3) enzyme plays important roles in abiotic stress responses^21^. In this study, we explored currently unidentified functions of ABA3 under stress. Mutations in the *ABA3* gene reduce endogenous ABA content, as do mutations in other ABA biosynthesis genes, such as *ABA1* and *ABA2*^20,38^. ABA1 (zeaxanthin epoxidase) and ABA2 [xanthoxin dehydrogenase, also referred to as short-chain dehydrogenase/reductase (SDR)], as well as ABA3, are each encoded by a single gene. ABA1 is involved in the xanthophyll cycle in addition to ABA biosynthesis, whereas additional functions of ABA2 other than ABA biosynthesis have not been reported. Thus, we compared genome-wide gene expression between *aba3* (*aba3-1*), *aba2* (*aba2-2*) and wild-type (WT) plants to distinguish genes that are regulated by ABA3, both dependent and independent of ABA. A public microarray database (eFP browser, http://bar.utoronto.ca/efp/cgi-bin/efpWeb.cgi ^39^); indicated that *ABA3* mRNA levels were rapidly increased within 3 h of treatment with polyethylene glycol (PEG) solution, which induces osmotic stress, and the levels were sustained for up to 24 h (Supplementary Fig. S2). We therefore conducted microarray analysis with seedlings that were immersed in PEG solution (25%, *c*. −1.25 MPa) for 3 h to monitor the initial stages of the response, which we termed short-term response (Fig. 1b and Supplementary Fig. S3). In addition, to determine the roles of ABA3 during long-term stress responses, we analyzed the transcriptome after incubation of seedlings on low water potential (−1.2 MPa) agar plates^40^ for 8 h (Fig. 1b and Supplementary Fig. S3): it has been reported that endogenous ABA content and expression of ABA-responsive genes attained maximum levels after 8–10 h under similar conditions^40,41^. We confirmed that wild-type plants subjected to short-term and long-term stress accumulated greater than 10 times the level of ABA compared to plants from control treatments (Fig. 1b). Note that ABA levels in wild type plants were not equivalent between the two control conditions, possibly because different treatments were applied (liquid media for short-term stress and solid media for long term stress). Although we observed a slight increase in ABA content in stressed *aba3* and *aba2* mutant plants, the levels were much lower than those in wild-type plants in all conditions tested.

**Figure 2:**
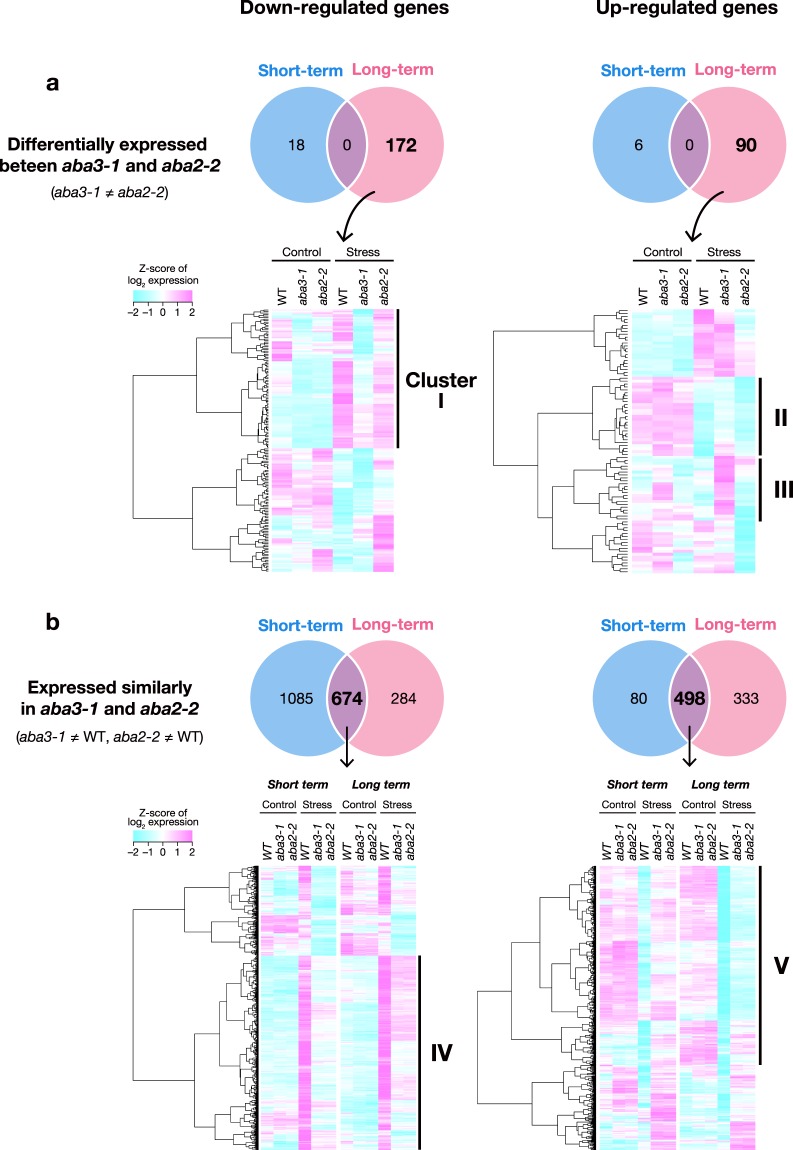
Genome-wide gene expression under short-term and long-term stress. (**a**) Genes that are differentially expressed between *aba3-1* and *aba2-2* plants in response to osmotic stress (*aba3-1* ≠ *aba2-2*). Numbers of genes whose expression levels were significantly different (±1.5 fold) between *aba3-1* and *aba2-2* plants under short-term or long-term stress are shown in the Venn diagrams (upper). The identified DEGs were classified into several clusters based on their expression in wild-type (WT), *aba3-1* and *aba2-2* plants by hierarchical clustering analysis (lower). (**b**) Genes that are expressed similarly in *aba3-1* and *aba2-2* plants but differentially between *aba3-1* and wild-type plants (*aba3-1* ≠ WT), and between *aba2-2* and wild-type plants (*aba2-2* ≠ WT). Numbers of genes whose expression levels were significantly different (±1.5 fold) in both *aba3-1* and *aba2-2* plants, compared to wild type, under short-term or long-term stress are shown in the Venn diagrams (upper). The identified DEGs were classified into several clusters based on their expression in wild-type (WT), *aba3-1* and *aba2-2* plants by hierarchical clustering analysis (lower).

We first selected genes that were differentially expressed (more than 1.5-fold) between *aba3* and *aba2* mutant plants under osmotic stress (Fig. 2). Only a small number of genes (18 and six down-regulated and up-regulated genes, respectively) were differentially expressed between *aba3* and *aba2* after the short-term stress treatment. In contrast, 262 genes (172 down- and 90 up-regulated genes) were identified as differentially expressed genes (DEGs) during the long-term stress treatment (Fig. 2a).

**Figure 3:**
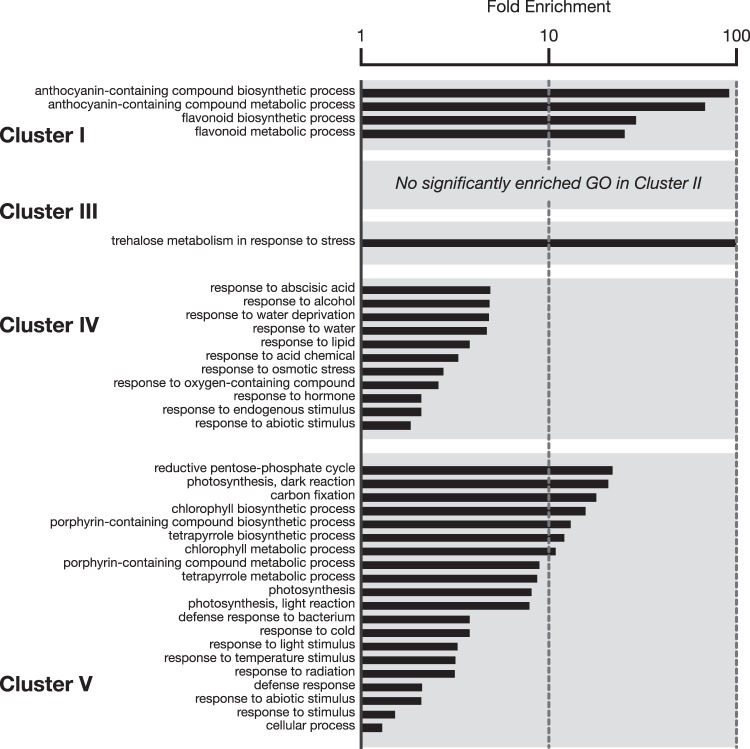
Functional classification of DEGs. DEGs in each cluster presented in Fig. 2 were classified according to the functional categories of the GO biological processes. Values are fold enrichment of each GO term (FDR, *P* < 0.05).

We then used hierarchical clustering analysis to classify the DEGs (independently for down- and up-regulated genes) from the long-term stress response into several groups according to their expression patterns in wild-type, *aba3* and *aba2* plants under both non-stress and stress conditions. Using this approach, we also identified genes that were differentially expressed between wild-type and *aba3* plants (Cluster I, II and III in Fig. 2a): these genes may require functional ABA3 but not ABA for their correct expression. Interestingly, GO enrichment analysis showed that in Cluster I, genes related to flavonoid and anthocyanin biosynthesis were enriched significantly (Fig. 3 and Supplementary Table S2). Cluster I is composed of 91 genes down-regulated specifically in *aba3* plant under stress. Expression of the genes in Cluster II were down-regulated by osmotic stress, however, the levels were relatively high in *aba3* compared with those in wild-type and *aba2* plants. Cluster III was composed of genes whose expression was not changed in wild-type plants under osmotic stress, but of genes whose expression was up-regulated in the *aba3* mutant in the presence and/or absence of osmotic stress. Although genes involved in trehalose metabolism were over-represented significantly in Cluster III, no significant enrichment in GO terms was observed for Cluster II.

We also identified genes that were differentially expressed in both *aba3* and *aba2* mutants when compared with wild-type plants (Cluster IV and V): these genes are potentially regulated in an ABA-dependent manner (Fig. 2b). As expected, GO enrichment analysis showed that genes involved in abiotic stress responses, including ABA-responsive genes, were over-represented in these clusters (Fig. 3). In total, more than 2900 genes were identified as DEGs under either short-term or long-term stress.

### ABA-dependent and -independent metabolomic responses to osmotic stress mediated by the ABA3 enzyme

To further explore the unknown functions of the ABA3 enzyme during stress responses, we performed metabolome analysis in addition to transcriptome analysis. The impact of the *aba3* mutation on the endogenous levels of more than 500 primary and secondary metabolites was investigated using a widely targeted metabolomics platform, as described in^42^. As a result, 19 compounds were found to be differentially accumulated (more than 1.5-fold) in *aba3* plants, compared to both wild-type and *aba2* plants, under short-term and/or long-term stress. These compounds were named differentially accumulated metabolites (DAMs) (Supplementary Fig. S4 and Supplementary Table S3). As expected from previous reports^22,29,43^, *aba3* plants under non-stress and stress (both short-term and long-term) conditions specifically accumulated high levels of xanthine, which is the substrate of XDH, whereas endogenous levels of allantoic acid, a downstream metabolite of xanthine, were greatly reduced. In addition, a purine metabolite, 3’,5’-cyclic guanosine monophosphate (cGMP), was accumulated at lower levels in *aba3* plants irrespective of the presence or absence of stress treatments. Interestingly, we also identified several compounds related to flavonoid and anthocyanin biosynthesis (shikimic acid, quercitrin, and quercetin-Rha-Glc-Rha) as DAMs. These results support the observation that genes related to flavonoid and anthocyanin biosynthesis were differentially expressed in *aba3* plants compared to wild-type and *aba2* plants.

### ABA3 has an important role in activating the later steps of the flavonoid biosynthesis pathway

**Figure 4:**
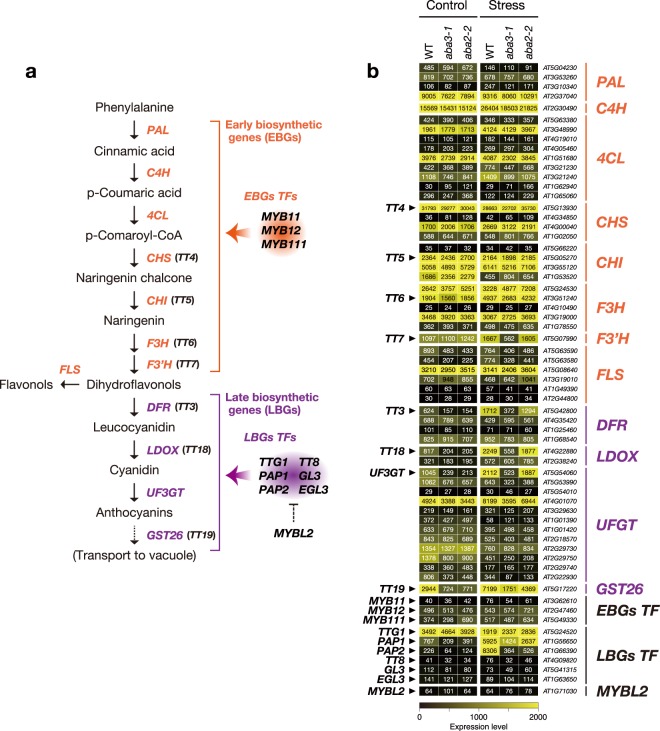
Effects of ABA3 disruption on flavonoid biosynthesis. (**a**) Flavonoid biosynthetic pathway. Orange and purple letters indicate the early biosynthetic genes (EBGs) and the late biosynthetic genes (LBGs), respectively. Genes encoding transcription factors (TFs) that regulate the flavonoid biosynthetic pathway are also shown on the right. (**b**) Expression of genes involved in flavonoid biosynthesis under long-term stress. Values are average signal intensities obtained from three independent microarray data experiments. PAL, Phenylalanine ammonia-lyase; C4H, Cinnamate 4-hydroxylase; 4CL, 4-Coumarate:CoA ligase; CHS, Chalcone synthase; CHI, chalcone isomerase; F3H, flavonol 3-hydroxylase; F3′H, flavonol 3′-hydroxylase; FLS, flavonol synthase; DFR, dihydroflavonol-4-reductase; LDOX, leucoanthocyanidin dioxygenase; UF3GT, UDP-glucose flavonoid-3-*O* glucosyltransferase; GST26, Glutathione S-transferase 26.

**Figure 5:**
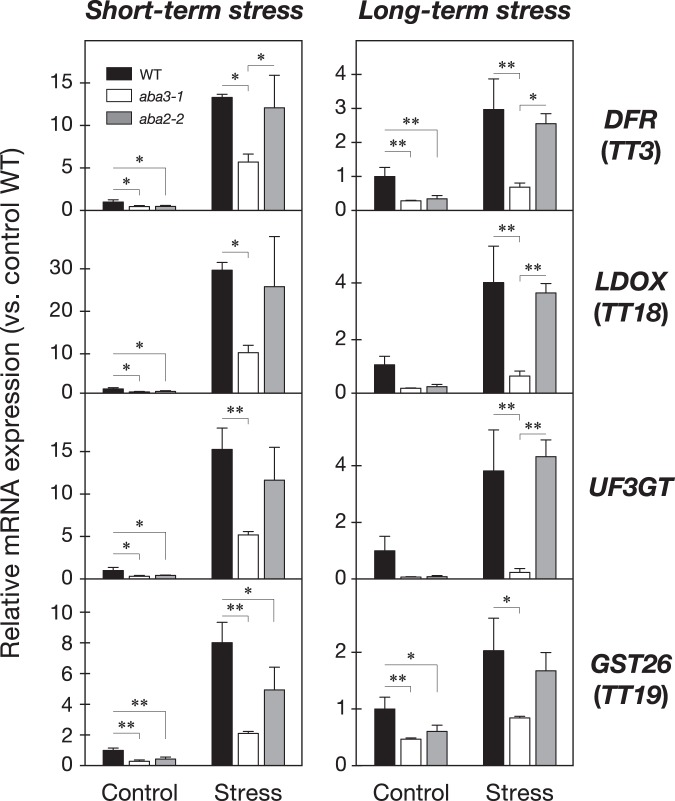
Expression levels of LBGs under short-term and long-term stress. Relative expression levels of four LBGs in wild-type (WT), *aba3-1* and *aba2-2* plants were analyzed by qRT-PCR. Values are means (±SD) of three biological replicates (^*^P < 0.05, ^**^P < 0.01 by Tukey’s multiple comparison test). DFR, dihydroflavonol-4-reductase; LDOX, leucoanthocyanidin dioxygenase; UF3GT, UDP-glucose flavonoid-3-*O* glucosyltransferase; GST26, Glutathione S-transferase 26.

Based on the results obtained from the transcriptome and metabolome analyses, we focused on the role of the ABA3 enzyme in the regulation of flavonoid biosynthesis during stress responses. The expression levels of 57 genes encoding 47 enzymes and 10 transcription factors, involved in the 10 reactions of the main flavonoid biosynthesis pathway determined by microarray analysis, were compared among wild-type, *aba3* and *aba2* plants before and after long-term stress treatment (Fig. 4a,b). Interestingly, we found that transcript levels of four TRANSPARENT TESTA genes *TT7*, *TT3*, *TT18* and *TT19*, which encode flavonoid 3’-hydroxylase (F3’H), dihydroflavonol 4-reductase (DFR), leucoanthocyanidin dioxygenase (LDOX), and glutathione S-transferase 26 (GST26), respectively, were reduced specifically in *aba3* plants. In addition, a gene encoding UDP-glucosyltransferase (UDP glucose-flavonoid 3-O-glucosyltransferase; UFGT) (*AT5G54060*), involved in glycosylation of flavonoids, was expressed at lower levels in *aba3* plants than in wild-type and *aba2* plants under stress (see the list of down-regulated DEGs in Supplementary Table S2). Further qRT-PCR analysis confirmed that the induction of four genes (*TT3*, *TT18*, *TT19* and *AT5G54060*) under short-term and long-term stress conditions, observed in wild-type and *aba2* plants, was reduced in *aba3* plants (Fig. 5). Interestingly, the four genes are located in the later steps of the flavonoid biosynthesis pathway and are known to play central roles in the production and accumulation of anthocyanins in vegetative tissues in response to environmental cues such as poor nutrition, high light, cold, salt, drought, and oxidative stress^44–47^.

### ABA3 contributes to anthocyanin accumulation under oxidative stress

**Figure 6:**
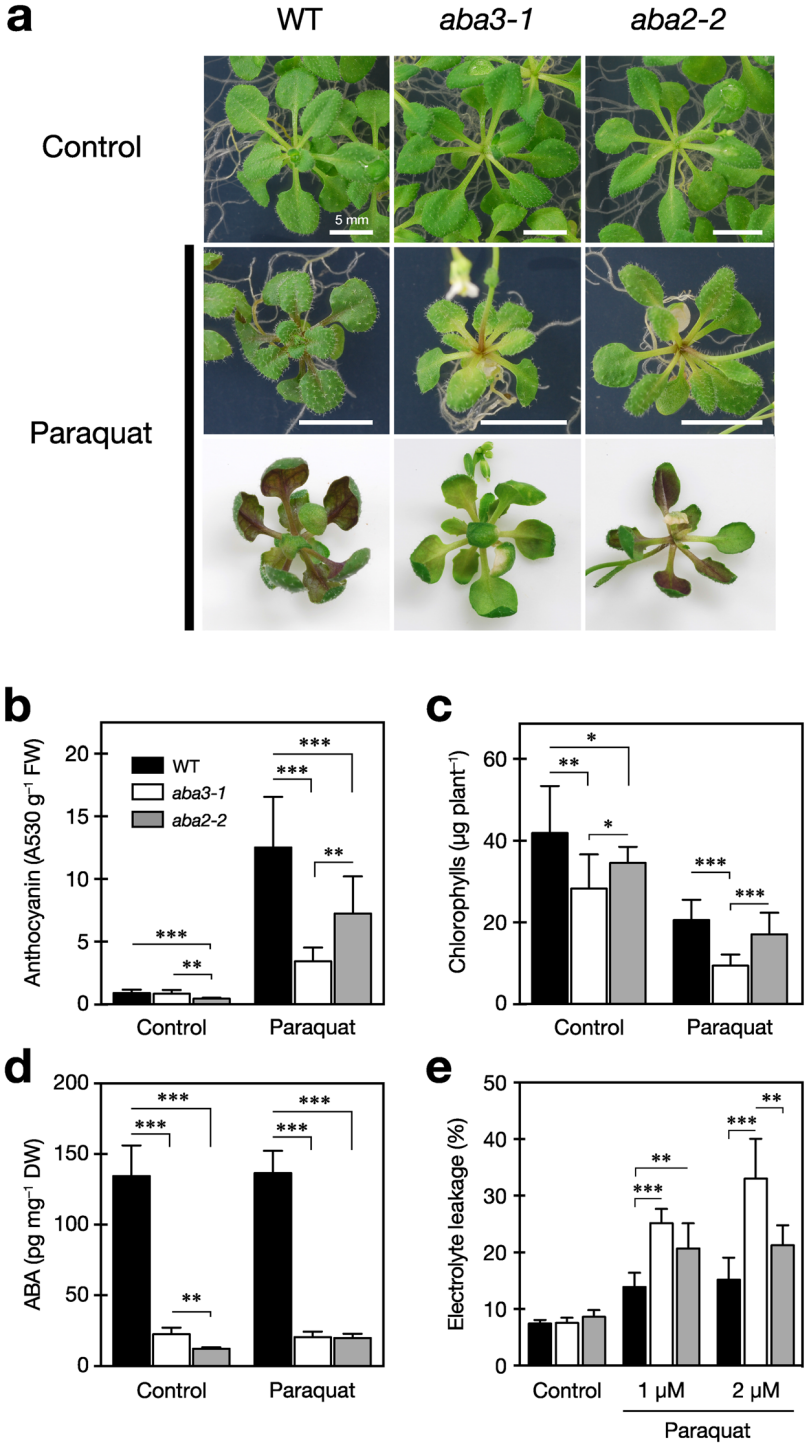
Reduced anthocyanin accumulation in *aba3-1* plants under oxidative stress. (**a**) Representative photos of wild-type (WT), *aba3-1*, and *aba2-2* seedlings after paraquat treatment. Twelve-d-old seedlings were transferred onto 1/2MS media containing 1 µM paraquat and photos were taken two weeks later (bars; 5 mm). (**b**) Anthocyanin contents in the aerial parts of wild-type (WT), *aba3-1*, and *aba2-2* seedlings after paraquat treatment. (**c**) Chlorophyll contents in the aerial parts of WT, *aba3-1*, and *aba2-2* seedlings after paraquat treatment. (**d**) ABA contents in the aerial parts of WT, *aba3-1*, and *aba2-2* seedlings after paraquat treatment. (**e**) Electrolyte leakage from the shoots of WT, *aba3-1*, and *aba2-2* seedlings after paraquat treatment. Values are the mean (±SD) of at least three biological replicates (^*^P < 0.05, ^**^P < 0.01, ^***^P < 0.001 by Tukey’s multiple comparison test).

Previous *in vitro* and *in vivo* studies have shown that flavonoid compounds, including their glycosides such as anthocyanins, have various bioactivities^48–52^. Of particular relevance to this study, it has been reported that anthocyanins exert antioxidant activities against ROS generated by stresses^53,54^. In Arabidopsis, it has been reported that oxidative stress markedly induced anthocyanin accumulation with the up-regulation of genes involved in the later steps of flavonoid biosynthesis, namely late biosynthesis genes (LBGs) such as *TT3* and *TT18* (Fig. 4a)^55^. Thus, we speculated that expression of LBGs under osmotic stress might be related to protection against oxidative stress. Since mRNA levels of LBGs were lower in *aba3* plants compared to wild-type and *aba2* plants under stress, we quantified endogenous levels of anthocyanins in wild-type, *aba3* and *aba2* plants that had been subjected to oxidative stress. After two weeks of oxidative stress treatment induced by paraquat, which is a well-known ROS generator, wild-type plants accumulated considerable amounts of anthocyanin pigments in their leaves (Fig. 6a,b). Anthocyanin levels in *aba2* plants were slightly lower than those in wild-type plants before and after paraquat treatment, suggesting that ABA promotes anthocyanin accumulation to some extent. However, interestingly, anthocyanins accumulated at much lower levels in *aba3* plants, compared with wild-type and *aba2* plants, after oxidative stress treatment. In addition to the reduced anthocyanin pigmentation, *aba3* plants exhibited a typical symptom of oxidative injuries, reduced chlorophyll content. This was significantly lower in *aba3* plants than in wild-type and *aba2* plants, especially under oxidative stress (Fig. 6c). Consequently, we determined cell membrane stability to estimate cellular damage caused by the stress and observed a higher rate of electrolyte leakage in *aba3* plants compared to that in both wild-type and *aba2* plants (Fig. 6e). Contrary to these *aba3*-specific phenotypes related to oxidative stress, the endogenous ABA content in *aba3* plants was reduced to a similar level to that of *aba2* plants, when compared with wild-type plants, in the presence or absence of oxidative stress (Fig. 6d). Finally, we confirmed that the phenotypes observed in *aba3* (*aba3-1*) plants were not allele-specific phenomena (Supplementary Fig. S5). These combined results suggest that the ABA3 enzyme regulates anthocyanin accumulation and hence oxidative stress tolerance independently from its role in ABA biosynthesis. Similarly, exogenous ABA application did not complement the *aba3*-specific phenotypes although the treatment enhanced paraquat-induced accumulation of anthocyanins (Supplementary Fig. S6a). It is also known that ABA3 is essential to produce allantoin, another bioactive small molecule involved in plant responses to abiotic stresses^30,35^. However, exogenous application of allantoin did not affect the phenotype observed in *aba3* plants, suggesting that this compound, like ABA, is also not involved in ABA3-specific stress responses (Supplementary Fig. S6b).

### AO and XDH are not involved in the ABA3-dependent accumulation of anthocyanins under oxidative stress

**Figure 7:**
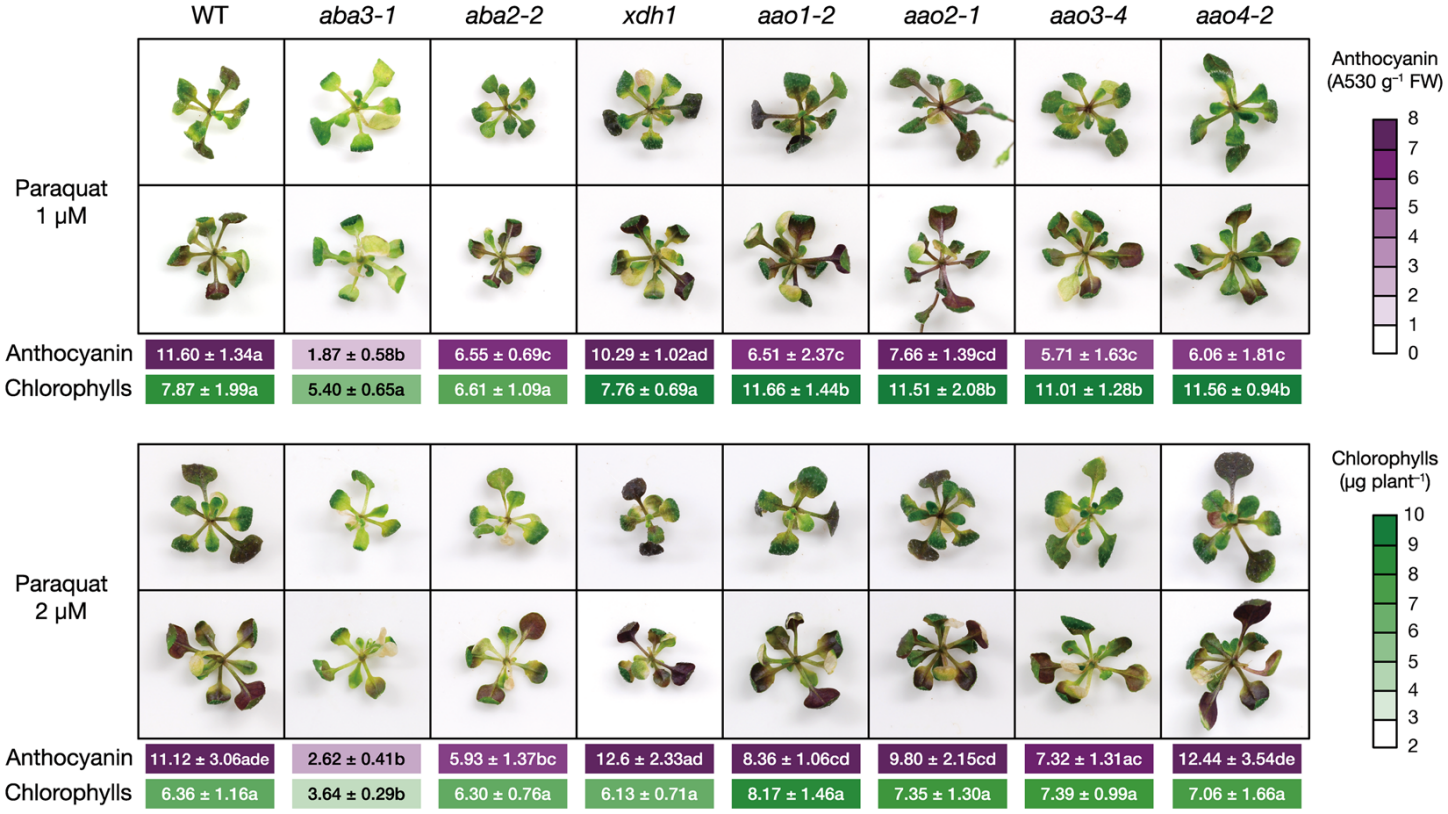
Anthocyanin and chlorophyll contents in mutants defective in Mo enzymes. Twelve-d-old seedlings of wild-type (WT), *aba3-1*, *aba2-2*, *xdh1*, *aao1-2*, *aao2-1*, *aao3-4*, and *aao4-2* were transferred onto 1/2MS medium containing 1 µM or 2 µM paraquat and anthocyanin and chlorophyll contents in the aerial parts were determined two weeks later. Representative photos of the mutant seedlings were also taken two weeks after paraquat treatment. Values are the mean (±SD) of three biological replicates. Different letters indicate statistically significant differences (*P* < 0.05) by Tukey’s multiple comparison test.

AO and XDH are enzymes that require sulfurated MoCo, which is produced by the reaction catalyzed by MOCOS (encoded by *ABA3* in Arabidopsis), as a prosthetic group^22^. There are four and two genes encoding AO and XDH, respectively, in Arabidopsis. In addition to its role in ABA biosynthesis, AO catalyzes the oxidation of a variety of aldehydes present in plants. The loss of XDH function not only results in the reduction of allantoin levels but also has a more global effect on purine metabolism. Therefore, we hypothesized that additional functions of AO or XDH might be involved in the ABA-independent oxidative stress responses mediated by the ABA3 enzyme. Consequently, we investigated the effects of oxidative stress on mutants defective in each of four AOs (*aao1*, *aao2*, *aao3* and *aao4*) and in the major XDH isoform, XDH1 (*xdh1*) (Leon-Kloosterziel, *et al*.^20^ Watanabe, *et al*.^35^ Nambara, *et al*.^56^ Seo, *et al*.^57^ and Supplementary Fig. S1). We found that all these mutants contained significantly higher levels of anthocyanins and chlorophylls than *aba3* plants after paraquat treatment (Fig. 7). These results suggest that ABA3 plays a role in anthocyanin accumulation in response to oxidative stress or in protection of chlorophyll from oxidative stress even in the absence of AO and XDH.

## Discussion

It is well known that the Arabidopsis ABA3 enzyme plays an essential role in ABA biosynthesis because the enzyme (AO) that catalyzes the last step of ABA biosynthesis requires sulfurated MoCo, synthesized by ABA3, for its enzymatic activity^25^. It was also reported recently that XDH, whose activity also depends on sulfurated MoCo, affects ABA accumulation indirectly^35^. In the present study, we first conducted transcriptome analyses using wild type, *aba3* and a typical ABA-deficient mutant *aba2* to elucidate currently unidentified ABA-independent physiological functions of ABA3. ABA2 is a member of the SDR family, which is composed of 56 gene products in Arabidopsis, however, the enzyme specifically involved in ABA biosynthesis appears to be encoded by a single gene (*ABA2*)^58^. Although ABA3 is also encoded by a single gene, endogenous ABA levels in *aba3-1* plants were slightly higher than those in *aba2-2* plants (Fig. 1b). It is possible that a shunt pathway *via* abscisic alcohol contributed to the accumulation of ABA in *aba3-1* plants to some extent^59^. Comparison of the transcriptome between wild-type, *aba3* and *aba2* plants revealed that many genes were commonly up- and down-regulated by osmotic stress in *aba3* and *aba2* plants compared to wild type (Fig. 2b). This suggests that reduced endogenous ABA levels in *aba3* and *aba2* plants had a significant impact on global gene expression under the stress conditions studied here. Correspondingly, genes that are related to ABA and/or stress responses were significantly enriched in the gene clusters (Fig. 3). Nevertheless, we also identified genes whose expression was differentially regulated between *aba3* and *aba2* plants under osmotic stress (Fig. 2a). These genes may be regulated in an ABA3- or ABA2-dependent manner. Further hierarchical clustering analysis identified at least three gene clusters (Cluster I, II and III) whose expression was deregulated in *aba3* plants alone. Interestingly, Cluster I, which contained genes whose expression was induced by osmotic stress in wild-type and *aba2*, but not in *aba3* plants, was enriched in GO terms related to the biosynthesis and metabolism of flavonoids and anthocyanins (Fig. 3). The main flavonoid biosynthesis pathway is divided into two parts, early and late (Fig. 4a). Among 34 genes encoding the early biosynthesis pathway enzymes, only one gene (*TT7*) was clearly expressed at a lower level in *aba3* plants than in wild-type or *aba2* plants under stress (Fig. 4b). In contrast, under the same stress, four out of 19 LBGs were down-regulated in *aba3* plants but not in *aba2*, compared to wild-type plants (Fig. 4b). Lower expression of the gene encoding a MYB-type transcription factor (TF), PRODUCTION OF ANTHOCYANIN PIGMENT (PAP) 1, in *aba3* plants may account for this observation, since PAP1 regulates the expression of LBGs by forming a protein complex composed of a bHLH-type TF [GLABRA3 (GL3), ENHANCER OF GLABRA3 (EGL3) or TT8] and a WD40-type TF (TTG1) (Fig. 4a)^60^. Consistent with these observations, several compounds related to flavonoid and anthocyanin biosynthesis were differentially accumulated in *aba3* plants compared to wild-type and *aba2* plants (Supplementary Fig. S4 and Supplementary Table S3). In contrast, expression of a *PAP1* homologue, *PAP2*, was similarly reduced both in *aba3* and *aba2* plants compared to wild type (Fig. 4b), suggesting that anthocyanin biosynthesis is also regulated by ABA. In fact, it has been reported that endogenous anthocyanin levels increase in response to various stresses and that several mutants defective in ABA biosynthesis or signaling contained reduced anthocyanin levels compared to wild type^55,61,62^.

It is well known that osmotic stress induces ROS production and hence oxidative stress^63^. It is also known that anthocyanins have antioxidant activities *in vitro* and act as a ROS scavenger in plants exposed to osmotic or oxidative stress^53–55^. Thus, although it is possible that the oxidative damage given to the plants was not equivalent between the PEG treatment and paraquat treatment, we speculated that ABA3 might induce the anthocyanin production in response to ROS accumulation, which leads to protection of the cells from oxidative damage. Our present study showed that the increase in anthocyanin levels in response to oxidative stress, which is normally observed in wild-type plants, was diminished in *aba3* plants (Fig. 6a,b). Interestingly, however, endogenous ABA levels in wild type did not increase in response to oxidative stress (Fig. 6d), suggesting that basal levels of ABA present in non-stressed plants affected the stress-induced accumulation of anthocyanins. In contrast, although *aba2* plants accumulated significantly higher levels of anthocyanins compared to *aba3* plants under oxidative stress, ABA levels in *aba3* plants were slightly higher or comparable to those in *aba2* plants (Fig. 6d). These results support the hypothesis that anthocyanin accumulation is regulated partly by the ABA3 enzyme, but independently from its role in ABA biosynthesis. In addition to reduced anthocyanin accumulation, *aba3* plants exhibited a bleaching phenotype in the presence of paraquat, indicating that the mutant is indeed sensitive to oxidative stress (Fig. 6c).

One reason for the reduced anthocyanin levels in *aba3* plants could be the down-regulation of *PAP1*. However, the mechanisms by which ABA3 regulates its expression are totally unknown. As mentioned above, it is unlikely that ABA could explain all the defects observed in *aba3* plants. This idea is supported by the observation that the reduced anthocyanin accumulation in *aba3* plants was not restored to the wild-type level by exogenous ABA application (Supplementary Fig. S6). One possible explanation is that the ABA3 enzyme is involved in the production of a bioactive compound that somehow induces anthocyanin accumulation, since MoCo synthesized by ABA3 is required for the activity of several enzymes. It has been reported that allantoin, which is an intermediate in the purine catabolism pathway, promotes the production of free bioactive ABA from its inactive glucose-conjugated form^35^. Mutants defective in XDH contained reduced allantoin levels and showed stress-sensitive phenotypes that can be partly explained by altered ABA levels. It is possible that allantoin has additional biological activities that enhance anthocyanin biosynthesis. Indeed, allantoin activates the JA response, and JAs play important roles in stress-induced accumulation of anthocyanins^32,64,65^. Endogenous levels of the bioactive hormone JA-Ile and its precursor JA were altered in *aba3* plants compared to wild-type plants (Supplementary Fig. S8). However, their levels were comparable between *aba3* and *aba2* plants, suggesting that JAs are also not involved in the ABA3-dependent accumulation of anthocyanins. In addition, exogenous application of allantoin did not rescue the *aba3*-specific reduction in anthocyanin levels (Supplementary Fig. S6). It is also known that xanthine-derived compounds other than allantoin, such as uric acid, have antioxidant activities^66^. However, the *xdh1* mutant accumulated anthocyanins at a similar level to wild-type plants under oxidative stress (Fig. 7). Some aldehyde compounds, which are potential substrates of AO, are also bioactive. Of these, α,β-unsaturated carbonyls, such as acrolein, are regarded as highly reactive aldehydes, named reactive carbonyl species (RCS). RCS function as signaling molecules, but excess amounts of aldehydes are cytotoxic due to their high electrophilicity, leading to protein modification and gene expression associated with stress responses^67–70^. It has been reported that AAO4 plays a key role in silique senescence by regulating cellular RCS levels during seed development^27^. However, anthocyanin levels in the *aao4* mutant plants, as well as in the *aao1*, *aao2* and *aao3* single mutants, were much higher than those in *aba3* plants (Fig. 7). Together, these data suggest that AO and XDH are not absolutely required for the ABA3-dependent regulation of anthocyanin biosynthesis. We speculate that there is an unidentified Mo enzyme, although we cannot exclude the possibilities that multiple AO and XDH isoforms regulate this process redundantly. Since *ABA3* is induced by osmotic stress and ABA (Xiong, *et al*.^21^ and Supplementary Fig. S2), it is possible that the loss of these enzymatic activities altered the ABA3-dependent (but AO and XDH-independent) stress responses. It is worth noting that the cyclic purine nucleotide cGMP, which is known to act as a second messenger like Ca^2+^, accumulated to a lesser extent in *aba3* plants compared to wild-type and *aba2* plants under stress and non-stress conditions (Supplementary Fig. S4b and Supplementary Fig. S7c). In several plant species, the involvement of cGMP and its nitrated derivative, 8-nitro-cGMP, has been reported in the regulation of several important physiological processes such as hormone signaling, ion transport, expression of stress-related genes, stomatal movement, and light response^47,71–79^. Interestingly, it was observed in cultured cells of soybean that the expression of anthocyanin biosynthesis genes was induced in response to exogenous cGMP application, resulting in an increased anthocyanin level^80^. Thus, it is possible that the lower anthocyanin levels in *aba3* plants are due to reduced levels of cGMP, although the mechanisms involved are unknown.

Our metabolome analyses showed that, in addition to anthocyanins, some compounds related to oxidative stress, such as GSH and pyrimidine precursors, differentially accumulated in *aba3* plants compared to wild-type and *aba2* plants under oxidative stress (Supplementary Fig. S7b,c). Cellular redox imbalance damages DNA, lipids and proteins and triggers ROS signaling and scavenging systems such as the ascorbate-GSH/GSSG cycle^81^. The central metabolites in pyrimidine biosynthesis, N-Carbamoyl-l-aspartate and orotate, are involved in electron transfer and redox balance in mitochondria^82,83^. These results suggest that the ABA3 enzyme plays important roles in oxidative stress responses thorough multiple processes. This idea is supported by the results from the multidimensional scaling analysis based on metabolite contents, in which *aba3* plants responded to oxidative stress in a very different manner to wild-type and *aba2* plants (Supplementary Fig. S7a). Currently, however, it is difficult to discriminate between the primary or direct effects caused by the loss of ABA3 functions and the accompanying secondary or indirect effects.

In conclusion, our study demonstrated the existence of an ABA-independent stress response pathway that is mediated by the ABA3 enzyme and suggested the presence of an unidentified Mo enzyme(s) that produces functional metabolite(s) involved in plant stress responses. We speculate that plants have evolved a system to regulate multiple metabolic pathways with a single enzyme (ABA3) to allow them to respond globally to, and to cope with, their ever-changing environment.

## Materials and Methods

### Plant materials and growth conditions

*Arabidopsis thaliana* (L.) Heynh. [accession Columbia-0 (Col-0)] was used as the wild type for all experiments in this study. Mutant lines used in the experiments were as follows: *aba3-1*^20^, *aba3-7* (SALK_054454)^84^, *aba3–8* (SAIL_576_D01), *aba2-2*^56^, *xdh1* (SALK_148366)^35^, *aao1-2* (SALK_069221)^26^, *aao2-1* (SALK_104895; Supplementary Fig. S1), *aao3-4* (SALK_072361)^57^, and *aao4-2* (SALK_057531)^27^. Homozygous *aba3-8* and *aao2-1* mutants were selected by PCR using primer combinations designed by the T-DNA Primer Design Tool (http://signal.salk.edu/tdnaprimers.2.html).

After surface sterilization with 70% (v/v) ethanol and then with 5% (v/v) NaClO (0.25% active chlorine) containing 1% (w/v) SDS, seeds were sown on half-strength Murashige-Skoog (1/2MS) media with 1% (w/v) sucrose and 0.8% (w/v) agar. To break dormancy, the seeds were incubated at 4 °C for 3 d in the dark, and the plates were placed in growth chambers at 22 °C under long-day conditions (16 h : 8 h, light : dark, 100 µmol photons m^−2^ s^−1^).

For osmotic stress treatments, 10-d-old seedlings were floated on MES buffer (pH 5.7) containing 25% (w/v) PEG for 3 h (short-term stress) or placed on PEG-infused 1/2MS plates^40^ for 8 h (long-term stress). MES buffer and 1/2MS plates were used as controls for the short-term and long-term stress treatments, respectively. For oxidative stress treatments, 12-d-old seedlings were transferred to 1/2MS plates supplemented with 1 µM or 2 µM paraquat and incubated for two weeks under continuous light.

### Microarray and qRT-PCR analyses

Total RNA was prepared from whole seedlings using the PureLink Plant RNA reagent (Thermo Fisher Scientific, Waltham, USA) according to the manufacturer’s instructions. For microarray analysis, the quality of RNA was checked with an Agilent 2100 Bioanalyzer (Agilent Technologies, Santa Clara, USA). Cy3-labeled complementary RNA (cRNA) probes were synthesized from 400 ng total RNA using the Quick Amp labeling kit (Agilent Technologies). The probes were hybridized to a custom Agilent 8 × 60 k array using the Gene Expression Hybridization kit (Agilent Technologies). Microarray chips were scanned using a Microarray scanner G2505B (Agilent Technologies). Expression levels were normalized by the Robust Multiarray Average (RMA) algorithm provided by the LIMMA package of the Bioconductor project and further analyzed using R statistical software ver. 3.4.0 (R Core Team, Vienna, Austria) and RStudio ver. 1.0.143 (RStudio Team, Boston, USA). Experiments were performed in triplicate, and differentially expressed genes (DEGs) between *aba3-1* and *aba2-2* mutants were identified by one-way ANOVA with the Benjamini-Hochberg False Discovery Rate (BH-FDR) controlled at 1% followed by the Student’s *t*-test with the BH-FDR controlled at 5%. Hierarchical clustering analysis with Euclidean distance and Ward linkage, and heat map visualization was performed using the gplots package^85^ in the R software. Gene ontology (GO) analysis was performed on the Gene Ontology Consortium web site (http://www.geneontology.org)^86^. All microarray data have been deposited in the Gene Expression Omnibus (GEO) database under the accession number GSE110079. For qRT-PCR analysis, total RNA was reverse-transcribed using the ReverTra Ace qPCR RT Master Mix with the gDNA Remover kit (Toyobo, Osaka, Japan) to generate cDNA. Quantification of mRNA levels was performed using the Mx3000P QPCR System (Agilent Technologies). All PCR amplifications contained 1 x SYBR Green Master Mix (THUNDERBIRD SYBR qPCR Mix; Toyobo), 0.2 µM reverse and forward primers and cDNA in a total volume of 20 µl. The sequences of primers used in this study are presented in Supplementary Table S1. Expression levels were normalized with the levels of 18S rRNA.

### Metabolome analysis

Widely targeted metabolome analysis using ultra-performance liquid chromatography coupled with tandem mass spectrometry (UPLC-MS/MS) was performed as described previously^42^. Samples were extracted with 80% (v/v) methanol containing 0.1% (v/v) acetic acid with internal standards, and targeted metabolites were separated and detected on a Xevo TQ-S LC-MS system (Waters, Mississauga, Canada). Metabolites whose endogenous levels were significantly altered in *aba3-1* plants compared with *aba2-2* plants were identified by one-way ANOVA with the BH-FDR controlled at 1% followed by the Student’s *t*-test with the BH-FDR controlled at 5%. Multidimensional scaling analysis based on metabolite contents was conducted using the R software.

### Measurement of anthocyanin and chlorophyll content

Endogenous levels of anthocyanins and chlorophylls were measured as described previously^87,88^ with some modifications. Briefly, the aerial parts of wild-type and mutant plants exposed to oxidative stress for two weeks were harvested and homogenized in 90% methanol using a TissueLyser II (Qiagen, Hilden, Germany). The extracts were mixed with an equal volume of 10% (v/v) acetic acid (for anthocyanins) or 10 x volume of 90% methanol (for chlorophylls). After centrifugation at 15,000 rpm for 10 min at 4 °C, the absorbance of supernatants was measured using the 96-well microplate reader SAFIRE (TECAN, Männedorf, Switzerland). Anthocyanin and chlorophyll contents were calculated according to formulae as previously described^87,88^.

### Hormone measurements

Endogenous ABA, JA, and JA-Ile were extracted with 80% (v/v) acetonitrile containing 1% (v/v) acetic acid from whole wild-type and mutant seedlings after freeze-drying. Hormone contents were determined using a UPLC-MS/MS system consisting of a quadrupole/ time-of-flight tandem mass spectrometer (Triple TOF 5600, SCIEX, Concord, Canada), and a Nexera UPLC system (Shimadzu Corp., Kyoto, Japan) as described previously^89^.

### Measurement of electrolyte leakage

Electrolyte leakage was measured as described previously^90^. Aerial parts from three wild-type and mutant seedlings were incubated in 5 ml sterilized deionized water for 4 h at room temperature and the conductivities of the solutions were measured with the electrical conductivity meter Twin Cond (HORIBA, Kyoto, Japan). The water containing the plant tissues was then autoclaved at 121 °C for 20 min and the conductivities of the solutions were measured. Electrolyte leakage of the samples (%) was calculated based on the conductivities after autoclaving.

### Statistical analysis

One-way ANOVA with the BH-FDR controlled at 1% followed by the Student’s *t*-test with the BH-FDR controlled at 5% was used to detect DEGs and DAMs. Significant differences among three or more groups were evaluated by Tukey’s multiple comparison test using the statistical software Prism 6 ver. 6 (GraphPad Software Inc., California, USA).

### Native PAGE and activity staining

AO activities were determined by in-gel activity staining after native PAGE as described previously^24^. Total protein was extracted from 10-d-old whole wild-type and mutant seedlings grown on 1/2MS media. Forty µg of protein was separated on 7.5% polyacrylamide gels without SDS, and then the gels were incubated at 30 °C for 60 min in buffer [100 µM potassium phosphate buffer (pH 7.5), 0.1 mM phenazine methosulfate, 0.4 mM 3(4,5-dimethylthiazolyl-2)2,5-diphenyltetrazolium bromide] containing 1 mM 2-naphthaldehyde as a substrate.

## Electronic supplementary material

Supplementary Information

Supplementary Table S2

Supplementary Table S4
