# Supplementary material for: Arabidopsis molybdenum cofactor sulfurase ABA3 contributes to anthocyanin accumulation and oxidative stress tolerance in ABA-dependent and independent ways
Source: Sci Rep. 2018 Nov 9;8:16592. doi: 10.1038/s41598-018-34862-1 (PMC6226459; doi:10.1038/s41598-018-34862-1)
Supplement: Supplementary file 1 — Supplementary Information [file 41598_2018_34862_MOESM1_ESM.pdf]

**Arabidopsis molybdenum cofactor sulfurase ABA3 contributes to anthocyanin accumulation and oxidative stress tolerance in ABA-dependent and independent ways**

Shunsuke Watanabe[1], Muneo Sato[1], Yuji Sawada[1], Maho Tanaka[1,2], Akihiro Matsui[1,2], Yuri Kanno[1], Masami Yokota Hirai[1], Motoaki Seki[1,2], Atsushi Sakamoto[3] and Mitsunori Seo[1,*]

[1]RIKEN Center for Sustainable Resource Science, 1-7-22 Suehiro-cho, Tsurumi-ku, Yokohama, Kanagawa, 230-0045, Japan

[2]RIKEN Cluster for Pioneering Research, 2-1 Hirosawa, Wako, Saitama, 351-0198, Japan

[3]Department of Mathematics and Life Sciences, Graduate School of Science, Hiroshima University, 1-3-1 Kagamiyama, Higashi-Hiroshima 739-8526, Japan

*Corresponding author: Mitsunori Seo, Email: mitsunori.seo@riken.jp

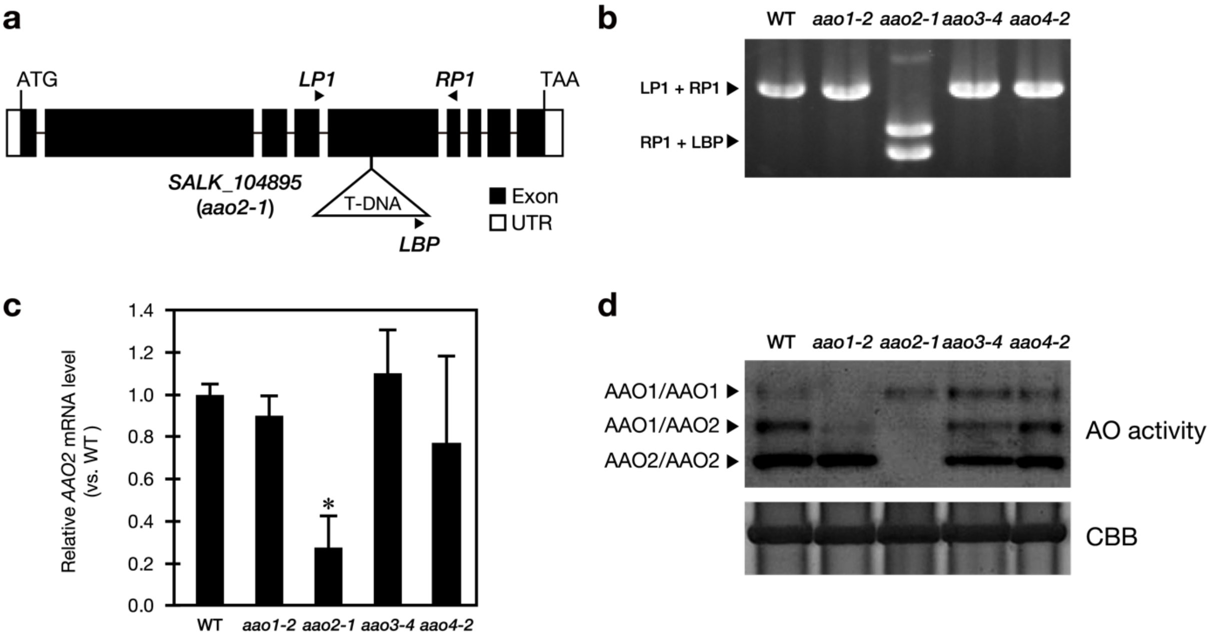


**Supplementary Figure S1. Characterization of *aao2-1* mutant.** (a) Structure of the *AAO2* gene and the site of T-DNA insertion in *aao2-1* (SALK_104895). Arrowheads indicate the positions of primers used for PCR-based genotyping. (b) Confirmation of homozygous T-DNA insertion in the *aao2-1* mutant by PCR. (c) Relative expression levels of *AAO2* in the *aao2-1* mutant. Total RNA was isolated from 10-d-old seedlings and used for qRT-PCR. Values are means (±SD) of three biological replicates. The asterisk indicates a significant difference compared to wild type by Tukey's multiple comparison test (*$P < 0.05$). (d) AO activities in *aao* mutants. The upper, middle and lower activity bands derive from homodimers of the AAO1 product, heterodimers of the AAO1 and AAO2 products, and homodimers of the AAO2 product, respectively. Major protein bands visualized by Coomassie brilliant blue (CBB) serve as the loading controls. The gel images were cropped around the region of interest and full-length gels are presented in Supplementary Figure S8.

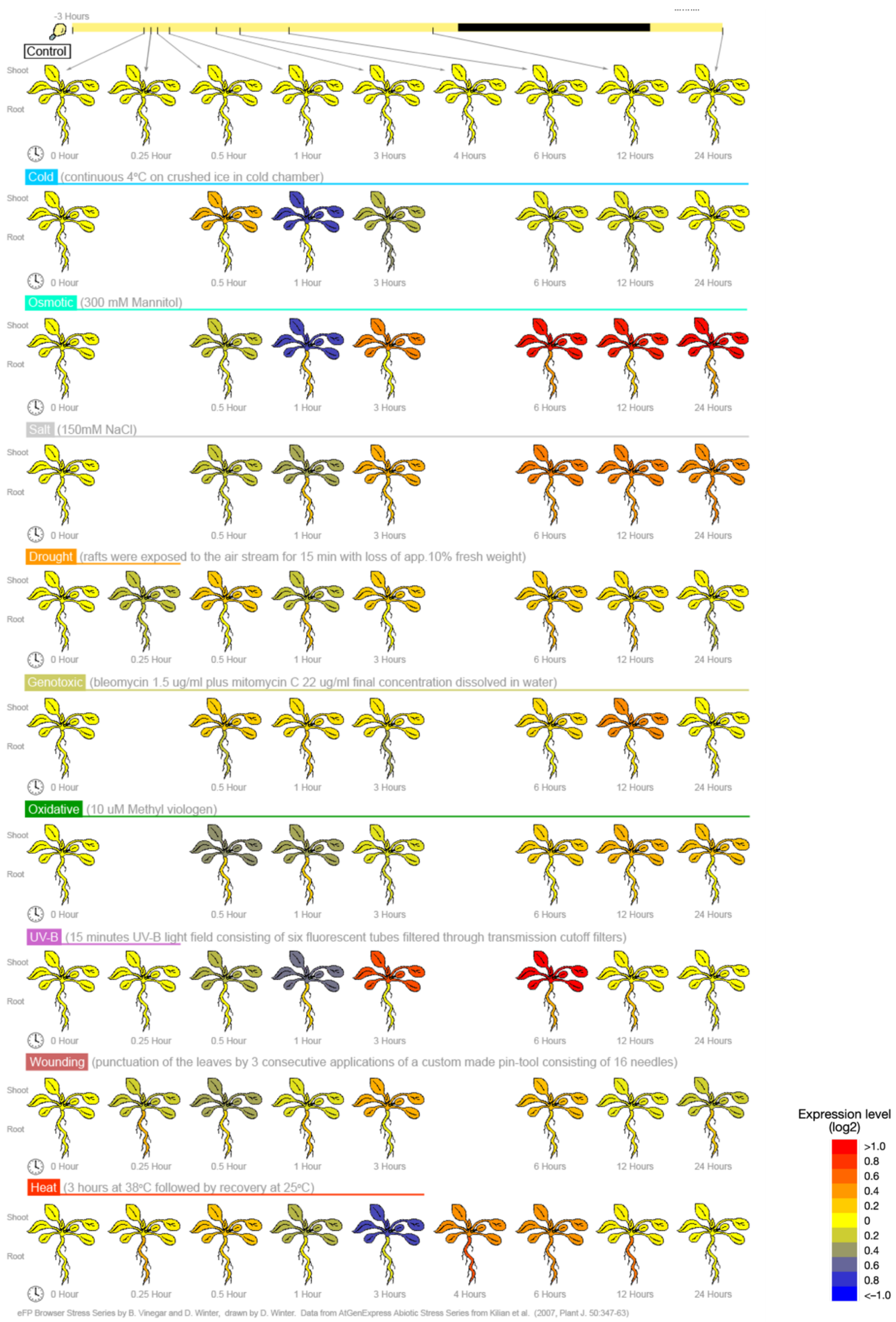


**Supplementary Figure S2. Expression levels of the *ABA3* gene under various abiotic stresses obtained from the Arabidopsis eFP Browser** (http://bar.utoronto.ca/efp/cgi-bin/efpWeb.cgi) (Winter et al. 2007 PLOS ONE 2:e718).

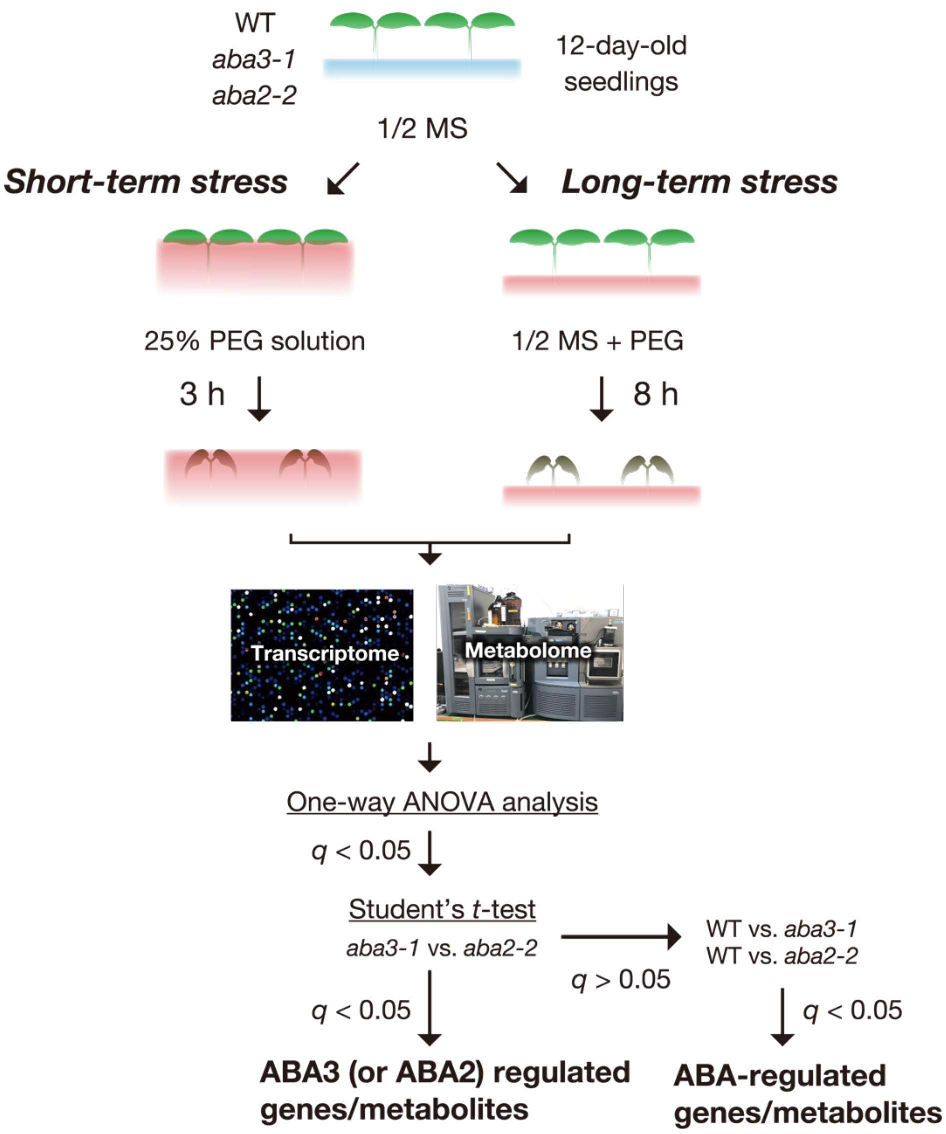


**Supplementary Figure S3. Comparative transcriptome and metabolome analyses with wild-type (WT), *aba2-2* and *aba3-1* plants to identify ABA3-regulated genes and metabolites.**

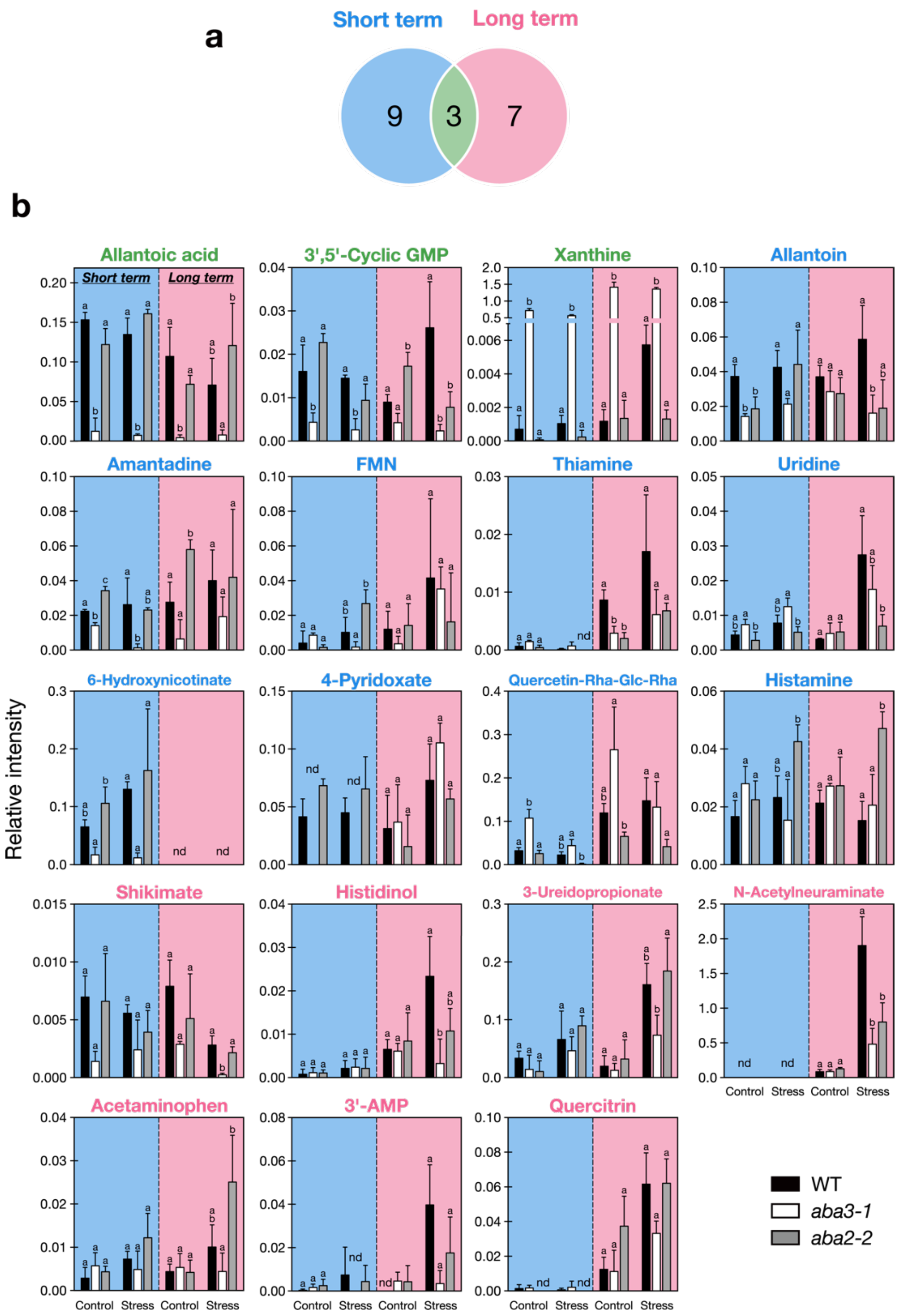


**Supplementary Figure S4. Differentially accumulated metabolites (DAMs) in the presence of osmotic stress.** (a) Numbers of metabolites that are differentially (±1.5 fold) accumulated in *aba3-1* compared to *aba2-2* and wild-type plants under short-term or long-term stress are shown in Venn diagrams.

**Supplementary Figure S4.** (*continued*) (b) Relative abundance of DAMs in *aba3-1*, *aba2-2* and wild-type (WT) plants. Values are means (±SD) of three biological replicates. Different letters indicate significant differences between genotypes within each treatment, except for the groups containing compounds not detected (nd), by Tukey's multiple comparison test ($P < 0.05$). Blue and pink backgrounds indicate short- and long-term stress, respectively. FMN, flavin mononucleotide.

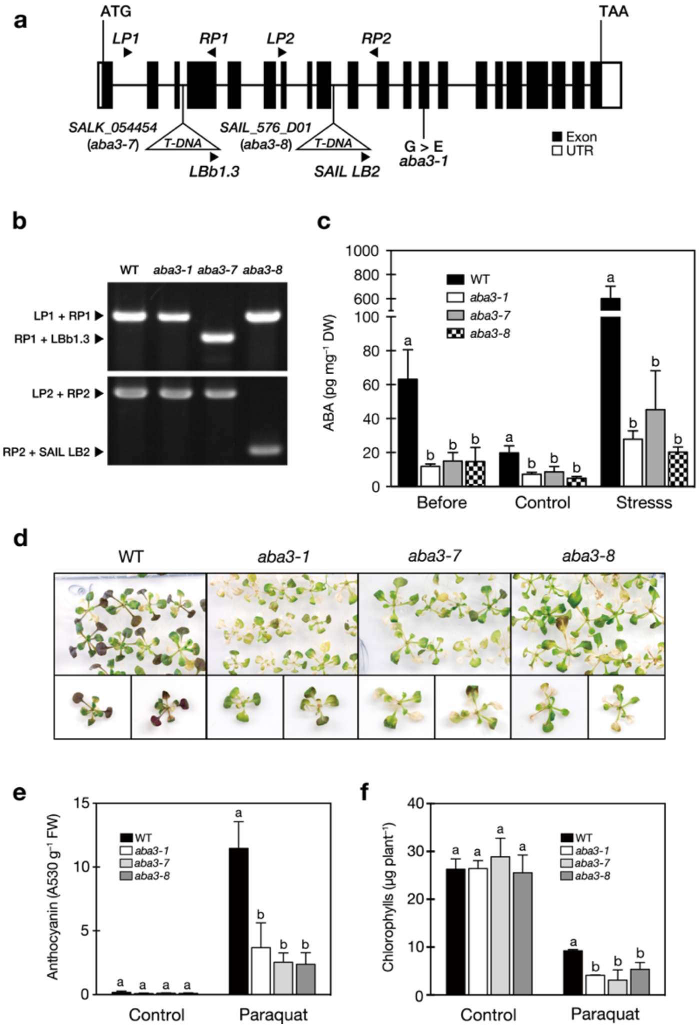


**Supplementary Figure S5**. **Phenotypes of *aba3* mutant alleles.** (a) Structure of the *ABA3* gene and mutations in three *aba3* alleles. Arrowheads indicate the positions of primers used for PCR-based genotyping of *aba3-7* (SALK_054454) and *aba3-8* (SAIL_546_D01). UTR, untranslated region. (b) Confirmation of homozygous T-DNA insertions by PCR. The gel images were cropped around the region of interest and full-length gels are presented in Supplementary Figure S8. (c) Endogenous ABA levels in *aba3* mutants before and after osmotic stress treatments (short-term stress treatment as shown in Fig. S2). For control treatments, seedlings were incubated in MES buffer without PEG. Values are means (±SD) of three biological replicates. Different letters indicate significant differences by Tukey's multiple comparison test (*$P < 0.05$). (d) Representative photos of wild type (WT) and *aao3* mutants that had been subjected to 2 μM paraquat treatment for two wk. (e) Endogenous anthocyanin levels in paraquat-treated *aba3* mutants. (f) Chlorophyll content in paraquat-treated *aba3* mutants. Different letters indicate significant differences ($P < 0.05$) by Tukey's multiple comparison test.

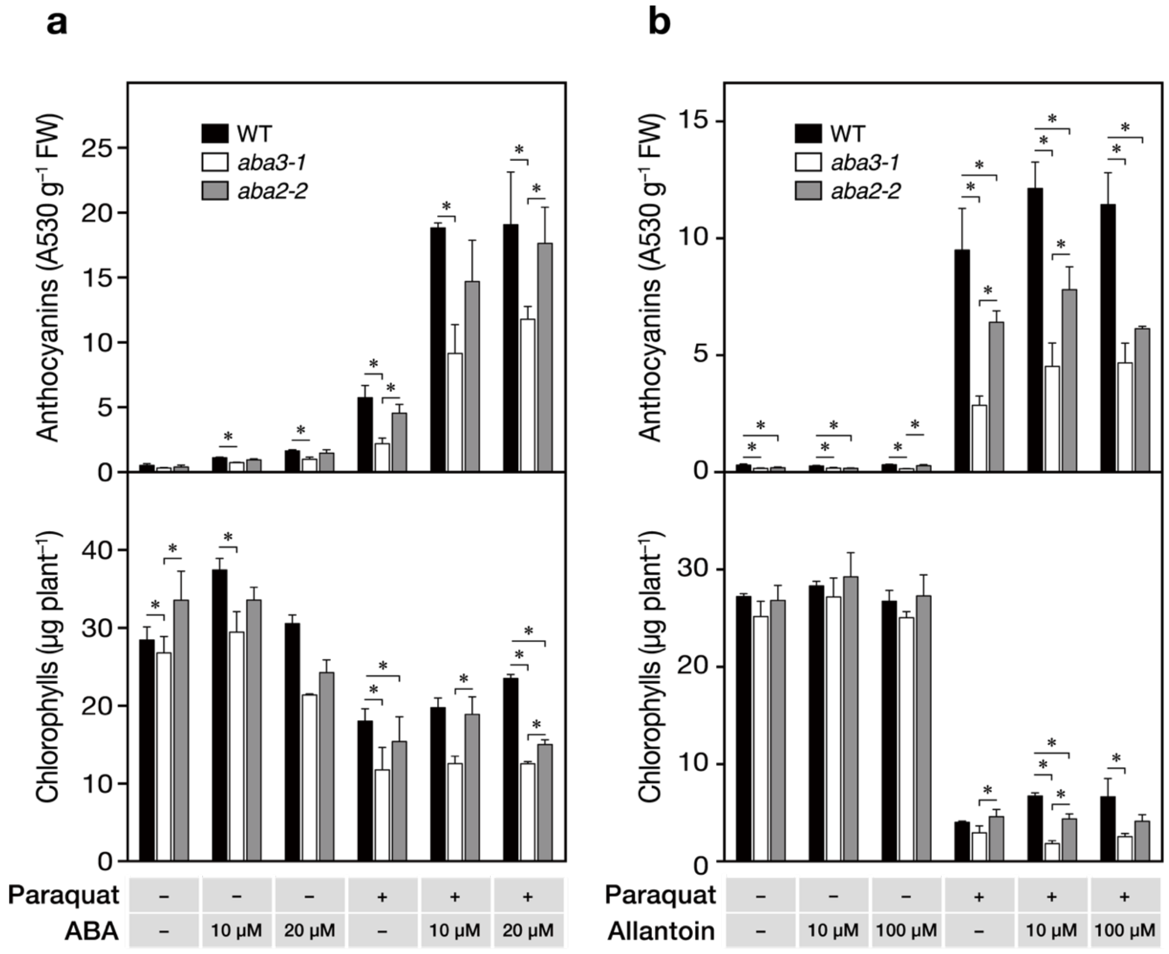


**Supplementary Figure S6. Effects of (a) ABA and (b) allantoin on anthocyanin and chlorophyll accumulation in response to oxidative stress.** Twelve-d -old seedlings were transferred to 1/2 MS media containing 2 µM paraquat plus ABA or allantoin. Concentrations of ABA and allantoin used for the experiments are indicated below the figures. Values are the mean (±SD) of three biological replicates (*$P$ < 0.05 by Tukey's multiple comparison test).

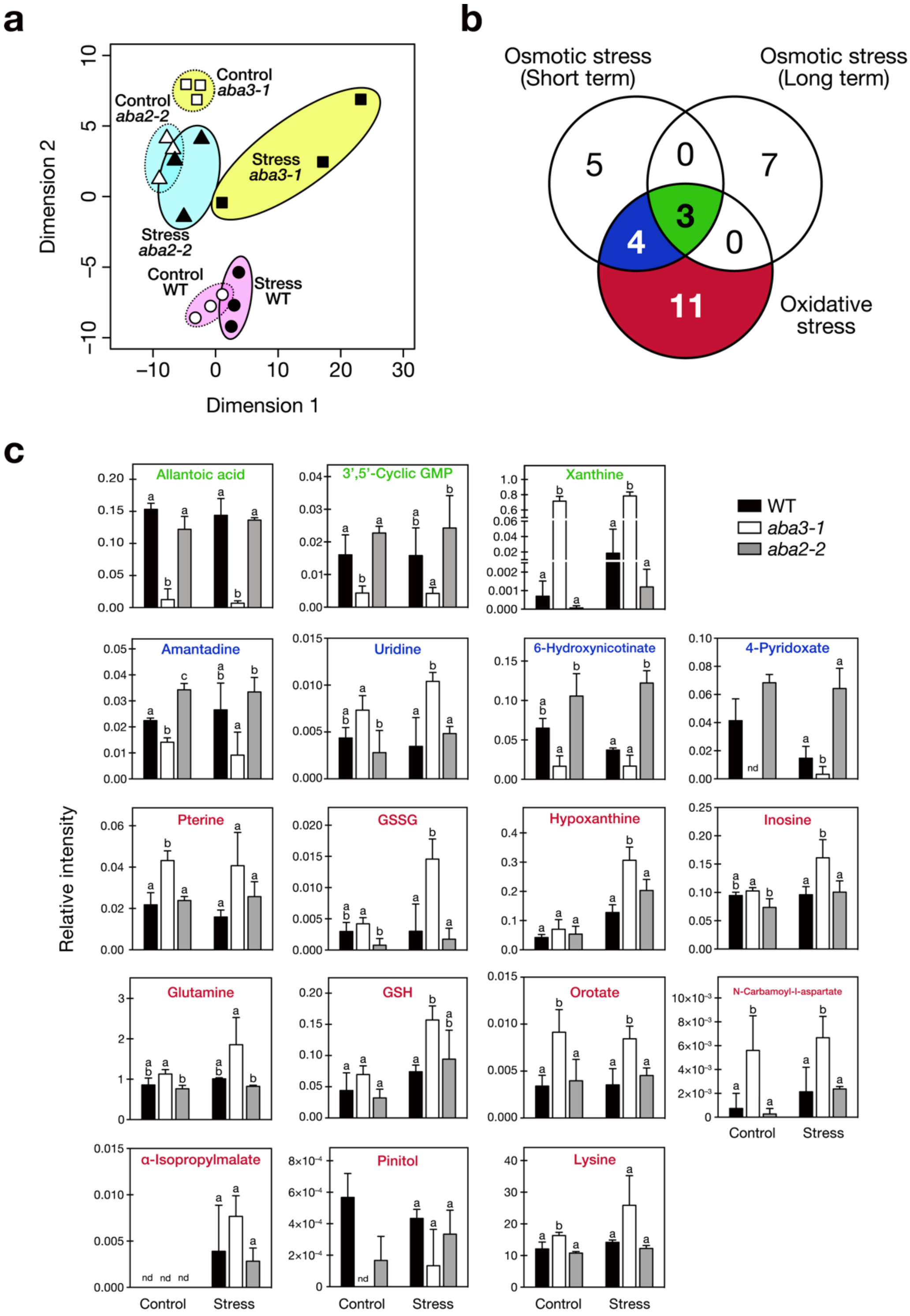


**Supplementary Figure S7. Changes in the metabolome in response to oxidative stress.** (a) Multi-dimensional scaling (MDS) analysis of metabolome data obtained from non-stressed (control) and stressed whole seedlings of wild type, *aba3-1*, and *aba2-2*.

**Supplementary Figure S7.** (*continued*) (b) Numbers of metabolites whose endogenous levels were significantly different (±1.5 fold) between *aba3-1* and *aba2-2* seedlings under short-term and long-term osmotic stress and oxidative stress are shown in a Venn diagram. Significant differences were analyzed by the Student's *t*-test controlling FDR at 5%. (c) Endogenous levels of metabolites that were accumulated differentially between *aba3-1* and *aba2-2* seedlings under oxidative stress. Values are means (±SD) of three biological replicates. Different letters indicate significant differences between genotypes within each treatment, except for the groups containing compounds not detected (nd), by Tukey's multiple comparison test ($P < 0.05$). GSH, reduced glutathione; GSSG, oxidized glutathione.

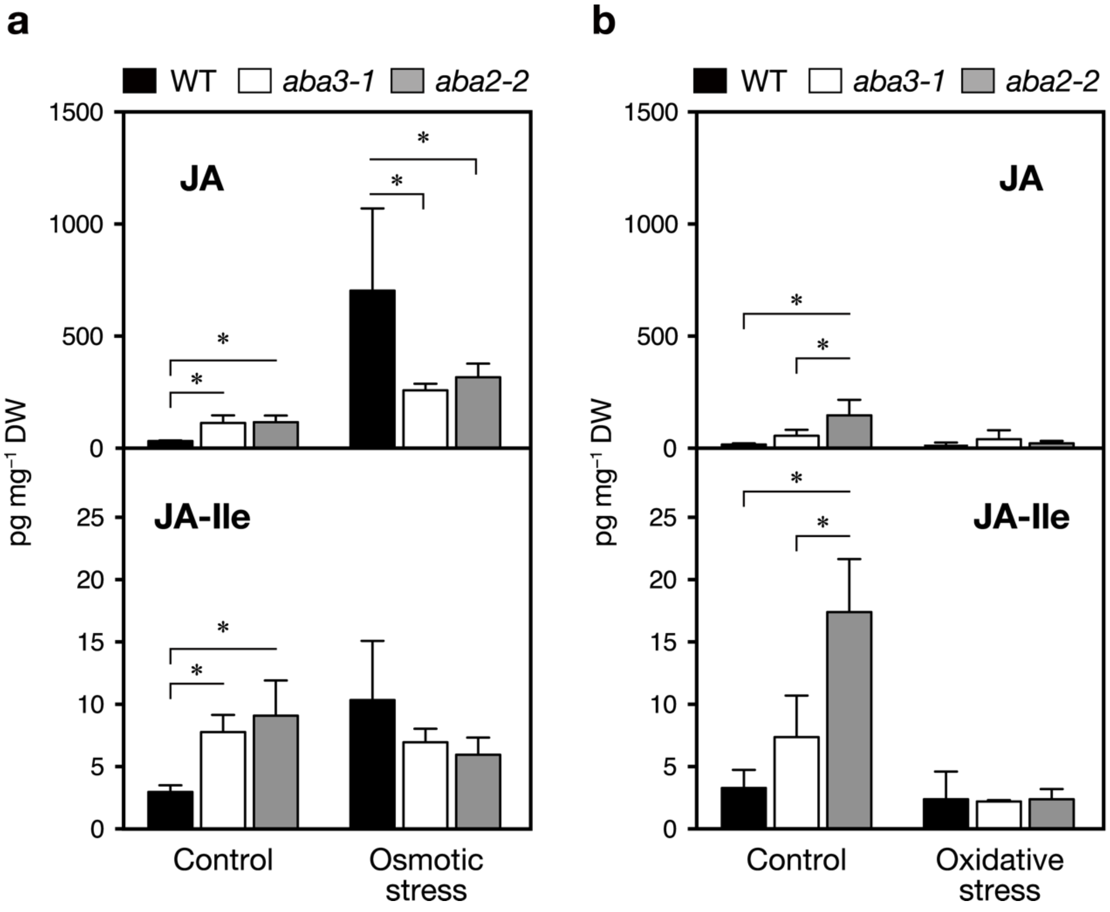


**Supplementary Figure S8. Endogenous levels of JA and JA-Ile in *aba3-1* and *aba2-2* plants under (a) osmotic stress (short-term) or (b) oxidative stress (2µM paraquat).** Values are means (±SD) of three biological replicates. Asterisks indicate significant differences by Tukey's multiple comparison test (*$P < 0.05$).

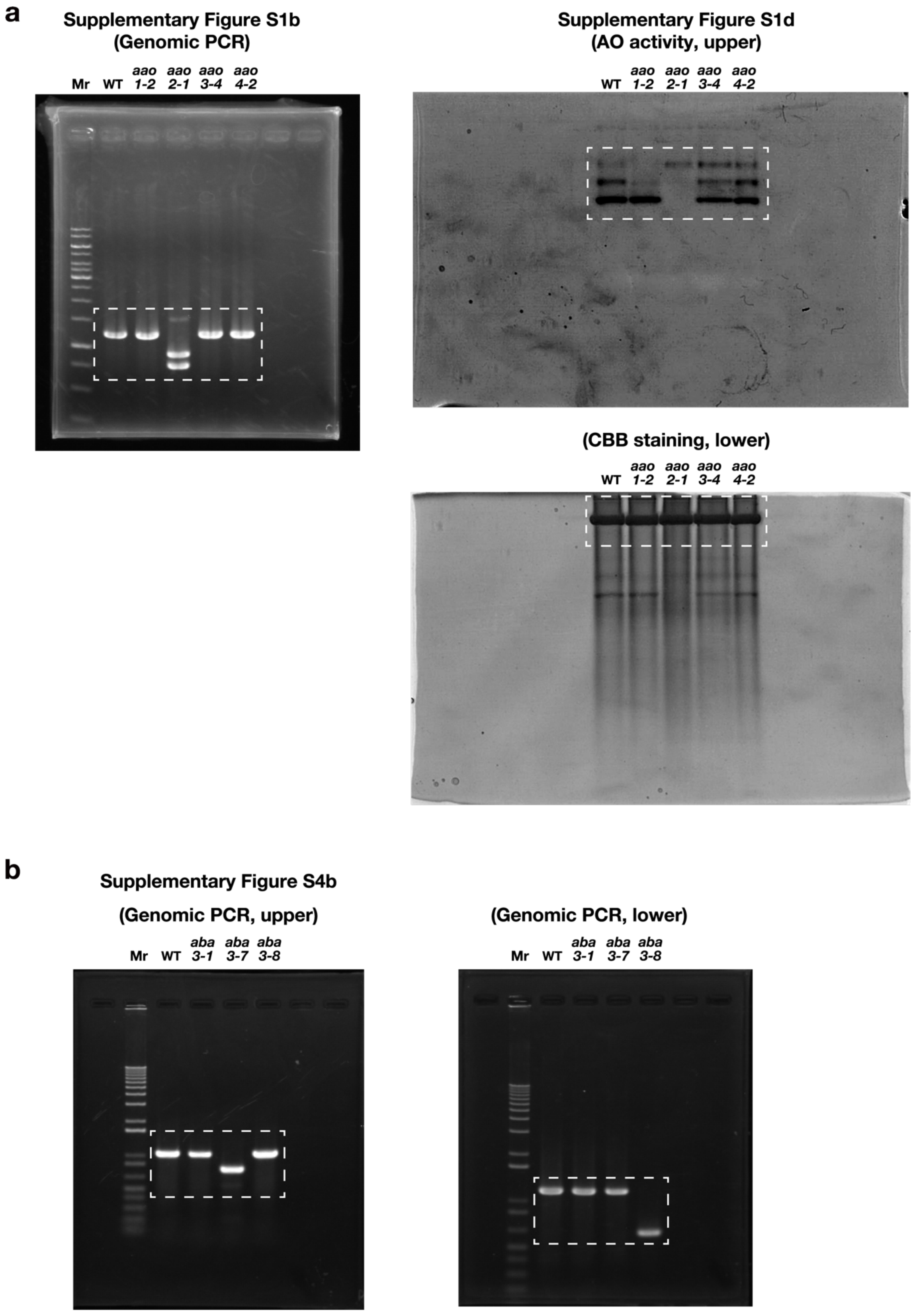


**Supplementary Figure S9. Full-length gels.** (a) Full-length gel corresponding in Supplementary Figure S1. (b) Full-length gel corresponding in Supplementary Figure S4. Dotted line boxes indicate the cropped images.

**Supplementary Table S1. Primers used in this study.**

| AGI | Gene symbol[a] | Direction | Primer sequence | Used for |
|---|---|---|---|---|
| At1g16540 | *ABA3* | Forward | 5’-GTGGATCAGCTCCACTCTCAC-3’ (LP1) | PCR genotyping |
| | | Reverse | 5’-TGTTTCTCATCTGCACAGCAC-3’ (RP1) | PCR genotyping |
| | | Forward | 5’-CTTTCTTGTTTTCGGCTGATG-3’ (LP2) | PCR genotyping |
| | | Reverse | 5’-TTGGGCCTGATTTATGTGAAG-3’ (RP2) | PCR genotyping |
| At3g43600 | *AAO2* | Forward | 5’-ACTGCATGGGAGTGTCTTTTG-3’ (LP) | PCR genotyping/ qRT-PCR |
| | | Reverse | 5’-GAGGTTTTGGAGGGAAATCTG-3’ (RP) | PCR genotyping |
| | | Reverse | 5’-ATATCGGTCTTGCGGTTGAC-3’ | qRT-PCR |
| At5g42800 | *DFR/ TT3* | Forward | 5’-TGGAGGTGTGATTTGGGTTT-3’ | qRT-PCR |
| | | Reverse | 5’-ACCGTTACAATCACACGCGA-3’ | qRT-PCR |
| At4g22880 | *LDOX/ TT18* | Forward | 5’-ACAAGAGGAATTGGTATCCGAGA-3’ | qRT-PCR |
| | | Reverse | 5’-AGGCACAAACACATCAAATTCA-3’ | qRT-PCR |
| At5g54060 | *UF3GT* | Forward | 5’-GCAAAGCTTGGAGAATGCTGT-3’ | qRT-PCR |
| | | Reverse | 5’-AGAGTCAGTCAAAACACATCTCCA-3’ | qRT-PCR |
| At5g17220 | *GST26/ TT19* | Forward | 5’-CCGTCTTGGAAGAAGCTTATGG-3’ | qRT-PCR |
| | | Reverse | 5’-AAAGCTTATTTGGTTCTTCAGATCA-3’ | qRT-PCR |
| - | *18S rRNA* | Forward | 5’-GCCCGGGTAATCTTTGAAAT-3’ | qRT-PCR |
| | | Reverse | 5’-GTACAAAGGGCAGGGACGTA-3’ | qRT-PCR |
| - | T-DNA | - | 5’-ATTTTGCCGATTTCGGAAC-3’ (LBb1.3) | PCR genotyping |
| - | T-DNA | - | 5’-GCTTCCTATTATATCTTCCCAAATTACCAATACA-3’ (SAIL LB2) | PCR genotyping |

[a] Gene symbols are provided by TAIR (http://www.arabidopsis.org/) except for T-DNA.

## Supplementary Table S3. Metabolites that were accumulated differentially between *aba3-1* and *aba2-2* plants under osmotic stress

| COMPOUNDS | KEGG LIGAND ID | METABOLISM/ BIOLOGOCAL PROCESS | Short-term osmotic stress | | | | | | | Long-term osmotic stress | | | | | | |
|---|---|---|---|---|---|---|---|---|---|---|---|---|---|---|---|---|
| | | | Relative metabolite level | | | | | | ANOVA *q*-value | Relative metabolite level | | | | | | ANOVA *q*-value |
| | | | Control (Fold change) | | | Stress (Fold change) | | | | Control (Fold change) | | | Stress (Fold change) | | | |
| | | | *aba3-1*/ WT | *aba2-2*/ WT | *aba3-1*/ *aba2-2* | *aba3-1*/ WT | *aba2-2*/ WT | *aba3-1*/ *aba2-2* | | *aba3-1*/ WT | *aba2-2*/ WT | *aba3-1*/ *aba2-2* | *aba3-1*/ WT | *aba2-2*/ WT | *aba3-1*/ *aba2-2* | |
| ***Short- and long-term stress*** | | | | | | | | | | | | | | | | |
| Allantoic acid | C00499 | Purine metabolism | 0.08 | 0.80 | 0.10 | 0.05 | 1.19 | 0.05 | $1.5 \times 10^{-6}$ | 0.04 | 0.67 | 0.06 | 0.11 | 1.70 | 0.06 | 0.009 |
| 3',5'-Cyclic GMP | C00942 | Purine metabolism | 0.27 | 1.42 | 0.19 | 0.18 | 0.65 | 0.27 | $4.8 \times 10^{-4}$ | 0.47 | 1.92 | 0.25 | 0.09 | 0.30 | 0.30 | 0.004 |
| Xanthine | C00385 | Purine metabolism | 1021.38 | 0.10 | 10724.50 | 543.26 | 0.23 | 2405.86 | $2.7 \times 10^{-10}$ | 1198.75 | 1.14 | 1053.24 | 236.86 | 0.23 | 1034.14 | $3.6 \times 10^{-10}$ |
| ***Short-term stress only*** | | | | | | | | | | | | | | | | |
| Allantoin | C01551 | Purine metabolism | 0.38 | 0.50 | 0.77 | 0.50 | 1.04 | 0.48 | 0.028 | 0.77 | 0.74 | 1.04 | 0.27 | 0.32 | 0.85 | 0.061 |
| Amantadine | C06818 | Unknown | 0.63 | 1.53 | 0.41 | 0.05 | 0.88 | 0.06 | 0.004 | 0.23 | 2.10 | 0.11 | 0.48 | 1.05 | 0.46 | 0.194 |
| FMN | C00061 | Riboflavin metabolism Oxidative phosphorylation | 2.13 | 0.39 | 5.49 | 0.17 | 2.64 | 0.07 | 0.006 | 0.30 | 1.18 | 0.26 | 0.85 | 0.39 | 2.16 | 0.584 |
| Thiamine (Vitamin B1) | C00378 | Thiamine metabolism | 2.20 | 0.65 | 3.38 | 5.25 | 0.00 | NC | 0.045 | 0.34 | 0.23 | 1.46 | 0.36 | 0.40 | 0.90 | 0.057 |
| Uridine | C00299 | Pyrimidine metabolism | 1.68 | 0.64 | 2.62 | 1.61 | 0.66 | 2.44 | 0.005 | 1.53 | 1.66 | 0.92 | 0.64 | 0.25 | 2.53 | 0.007 |
| 6-Hydroxynicotinate | C01020 | Nicotinate and nicotinamide metabolism | 0.26 | 1.63 | 0.16 | 0.09 | 1.25 | 0.07 | 0.028 | NC | NC | NC | NC | NC | NC | - |
| 4-Pyridoxate | C00847 | Vitamin B6 metabolism | 0.00 | 1.65 | 0.00 | 0.00 | 1.46 | 0.00 | 0.001 | 1.18 | 0.50 | 2.35 | 1.44 | 0.78 | 1.85 | 0.043 |
| Quercetin-Rha-Glc-Rha | - | Flavonoids | 3.35 | 0.80 | 4.21 | 1.96 | 0.06 | 35.57 | $2.0 \times 10^{-5}$ | 2.22 | 0.55 | 4.06 | 0.90 | 0.28 | 3.19 | 0.015 |
| Histamine | C00388 | Histidine metabolism | 1.68 | 1.35 | 1.25 | 0.66 | 1.83 | 0.36 | 0.049 | 1.28 | 1.28 | 0.99 | 1.35 | 3.10 | 0.44 | 0.011 |
| ***Long-term stress only*** | | | | | | | | | | | | | | | | |
| Shikimate | C00493 | Biosynthesis of phenylpropanoids | 0.20 | 0.95 | 0.21 | 0.71 | 0.94 | 0.75 | 0.501 | 0.37 | 0.65 | 0.56 | 0.09 | 0.77 | 0.11 | 0.020 |
| Histidinol | C00860 | Histidine metabolism | 1.42 | 1.33 | 1.06 | 0.60 | 1.00 | 0.60 | 0.974 | 0.94 | 1.29 | 0.72 | 0.14 | 0.46 | 0.30 | 0.044 |
| 3-Ureidopropionate | C02642 | Pyrimidine metabolism | 0.42 | 0.32 | 1.34 | 0.32 | 0.54 | 0.60 | 0.621 | 0.64 | 1.63 | 0.39 | 0.46 | 1.15 | 0.40 | 0.001 |
| N-Acetylneuraminate | C00270 | Amino sugar and nucleotide sugar metabolism | NC | NC | NC | NC | NC | NC | - | 1.01 | 1.48 | 0.69 | 0.25 | 0.42 | 0.60 | $3.3 \times 10^{-5}$ |
| Acetaminophen | C06804 | - | 2.00 | 1.51 | 1.32 | 0.40 | 0.90 | 0.44 | 0.806 | 1.22 | 0.96 | 1.27 | 0.44 | 2.49 | 0.18 | 0.015 |
| 3'-AMP | C01367 | Purine metabolism | 4.90 | 7.40 | 0.66 | NC | NC | 0.90 | 0.806 | NC | NC | 1.07 | 0.09 | 0.44 | 0.20 | 0.026 |
| Quercitrin | C01750 | Flavonoids | 1.30 | 0.00 | NC | NC | NC | NC | 0.700 | 0.90 | 3.01 | 0.30 | 0.54 | 1.01 | 0.53 | 0.007 |

'NC' indicates not calculated, as these metabolites were at levels below the detection limit in WT and *aba2-2* plants.
